# A Study of Emotional Intelligence Among Postgraduate Medical Students in Delhi

**DOI:** 10.7759/cureus.989

**Published:** 2017-01-22

**Authors:** Rajkrishna Ravikumar, O P Rajoura, Rahul Sharma, Manjeet S Bhatia

**Affiliations:** 1 Department of Community Medicine, University College of Medical Sciences and GTB Hospital; 2 Department of Psychiatry, University College of Medical Sciences and GTB Hospital

**Keywords:** emotional intelligence, medical students, doctors, postgraduate

## Abstract

Context: The importance of emotional intelligence (EI) in the successful practice of medicine has been well established. However, efforts to study the same in India, especially in doctors and medical students, are lacking. This study has measured the emotional intelligence of postgraduate medical students in Delhi and aimed to identify some of the factors affecting it.

Methods: A cross-sectional analytical study, using the Schutte's Self-Report Emotional Intelligence Test, to measure the EI of 200 postgraduate medical students of two medical colleges in Delhi. Students working in clinical specialties were selected randomly and contacted by the first author. Data was collected by a self-administered questionnaire.

Results: The mean scores of EI among the study participants was 124.4 with a standard deviation of 12.8. The total scores possible in the scale range from 33 to 165, with higher scores representing higher EI. The age of the participants was positively associated with emotional intelligence (r = 0.187, p = 0.008). EI was also found to decrease with the increase in total workload (p=0.013), having night duty hours (p = 0.019), and having emergency duty (p = 0.037).

Conclusions: More studies to measure the EI of doctors, the factors affecting it, and possible measures to improve it, are needed. The workload of the resident doctors needs to be assessed with appropriate changes made in the total work done and the quality of work done.

## Introduction

Emotional intelligence (EI) has been defined as “the ability to monitor one’s own and other people’s emotions, to discriminate between different emotions and label them appropriately, and to use emotional information to guide thinking and behavior” [1]. Emotional intelligence has the following common components or factors: perceiving, understanding, using, and managing emotions [1]. Perceiving as well as utilizing and managing emotions is essential to everyday practice for people in different careers - management professionals, business executives, doctors, advocates, and even students. Healthcare, as a whole, is not provided by an individual, but by a team. As the leader of the healthcare team, the doctor is expected to successfully manage both themselves as well as the rest of the team members in order to succeed as effective practitioners. Therefore, there has been a shift in thinking - from individual achievement to team achievement [2-3].

Research has shown a limited association between emotional intelligence and patient satisfaction [4]. Though there are considerably many factors associated with patient satisfaction, one among the important ones is the behavior of the doctor with the patient [5]. This can be focussed upon for improvement, if needed. Emotionally intelligent doctors are better able to perceive the need of the patient and are then able to address issues if any arise. In today’s world, where patient satisfaction is one of the most important criteria for a successful medical practice, emotional intelligence of doctors plays a vital role. Research has shown an increase in patient satisfaction scores after a period of emotional intelligence training of medical residents, as compared to previous scores [6]. 

In the case of medical students, emotional intelligence has been proven to be associated with their academic and clinical performance [7-12], as well as the amount of stress affecting their lives [13-18]. Students with low emotional intelligence have a higher risk of indulging in health-damaging behaviour in response to stress, along with it affecting their sleep habits. On the other hand, their emotionally intelligent counterparts were more likely to manage their stress using social support and were able to judge that such behaviour would not help [13].

Emotional intelligence also plays a role in communication skills [19-20], job satisfaction [4, 21], academic and clinical performance [7-12], alleviating stress [13-18] and burnout [4, 11, 18], and maintaining patient satisfaction [4, 6, 22-23] and a good doctor-patient relationship [22].

Research studies on EI are few in number in India, especially in postgraduate medical students. The purpose of this study is to understand emotional intelligence in the Indian context and to establish a score of EI, which could be used by future studies to interpret the various correlates of emotional intelligence as well as its implications in the medical profession.

## Materials and methods

This was a cross-sectional, analytical study. The units of study were 200 postgraduate medical students studying and working in the clinical departments of two randomly selected government medical colleges of Delhi and their affiliated hospitals. Postgraduate students directly involved in patient care were included in the study. Students having major psychiatric disorders, drug dependence, or those who were unable to be contacted after three separate visits were excluded. 

Written permission of the head of the institutions was obtained and a list containing the names of all the postgraduate medical students studying in each of the institutions was prepared. The study participants were selected by simple random sampling using a random number table. They were contacted personally, the purpose of the study was explained, and a good rapport was established. Sufficient time was spent with each participant to explain the purpose of the study, and doubts, if any, were clarified. An informed written consent was taken from those students who were willing to participate. The participants were requested to provide correct and complete information and were assured that the individual information collected would be kept strictly confidential.

Data collection was done using the following tools: 1) Demographic data was collected using a pre-tested semi-open-ended questionnaire. 2) Emotional intelligence was measured using the Schutte Self-Report Emotional Intelligence Test. It is a 33 item validated, self-reported measure of emotional intelligence developed by Schutte, et al. based on the EI model proposed by Salovey and Mayer. Participants’ response to each question is based on a 5-point Likert scale ranging from strongly disagrees to strongly agrees. The total score ranges from 33 to 165. The scale comprises four subscales that consist of: a) perception of emotion, b) managing one's own emotions, c) managing others’ emotions, and d) utilisation of emotions. The sum of these four subscales gives the total emotional intelligence score of the individual. In validation studies, the test demonstrated high internal consistency (Cronbach’s alpha = 0.90) and acceptable test-retest reliability (0.78) as well as excellent construct, predictive, and discriminant validity [24].

Ethical clearance was obtained from both the 1) Institutional Ethics Committee - Human Research (IEC-HR), University College of Medical Sciences, University of Delhi, Delhi and 2) Academic and Ethical Committee, Guru Teg Bahadur Hospital, Govt. of NCR of Delhi. Emotional intelligence scores were disclosed to the participants on demand.

Collected data was compiled, cleaned, and analysed using Statistical Package for Social Sciences (SPSS) software version 20.0 (IBM SPSS Statistics, Armonk, NY). Tests of significance (t-test, univariate analysis of variance (ANOVA), and correlation coefficient) were used to find the associations between emotional intelligence and its component factors and various demographic characteristics.

## Results

The demographic characteristics of the participants have been depicted in Table 1. The majority of students (164; 82%) were in the age group of 25 – 28 years, with the mean age of the study participants being 26 years. Almost 70% (139) of the postgraduate students were male. Most of the participants were single/unmarried (173; 86.5%) while the rest were married (27; 13.5%). A major proportion of the study participants stayed away from home in rented accommodations (99; 49.5%) or hostels (52; 26%). The rest of the students either stayed with family (44; 22%) or with local guardians (5; 2.5%).

**Table 1:** Work-related Characteristics of the Study Participants (n = 200)

Factor	Number (n = 200)	Percentage (%)
*A) Specialty*		
Medicine and Allied Specialties	108	54.0
Surgery and Allied Specialties	92	46.0
*B) Year of Study*		
First Year	68	34.0
Second Year	66	33.0
Final Year	66	33.0
*C) Total Hours Worked per Week*		
< 48 hours	32	16.0
48 - 96 hours	145	72.5
> 96 hours	23	11.5
*D) Night Duty Hours per Week*		
No Night Duty	25	12.5
12-23 hours	86	43.0
24 hours	45	22.5
> 24 hours	44	22.0
*E) Emergency Duty per Week*		
No Emergency Duty	24	12.0
12 - 23 hours	29	14.5
24 hours	80	40.0
> 24 hours	67	33.5

The study participants were pursuing their postgraduate studies in medicine and allied specialties (108; 54%) and surgery and allied specialties (92; 46%). Most of the resident doctors (145; 72.5%) worked for around 48-96 hours every week. Some of the students (23; 11.5%) worked for more than 96 hours per week while only some students (32; 16%) worked for less than 48 hours per week. A large number of students were reported to work for more than 24 hours of night duty and emergency duty per week while a few students did not have any night duty or emergency duty.

Out of the total number of participants, 35 (17.5%) reported having a source of significant stress in their life. Stress was mostly reported to be due to work or academics. Some students mentioned other sources of stress like family issues, personal problems, career difficulties and relationship problems.

The emotional intelligence scores of the postgraduate students ranged from 88 to 159, with a mean score and standard deviation of 124.4 (12.8) (Table 2). The distribution of scores for the subscales of EI has been depicted in Table 2. A statistically significant association was observed between the age and EI scores of the study participants. However, it may be noted that the correlation was weak. As the age of the students increased, the emotional intelligence was found to increase (r = 0.187, p = 0.008).

**Table 2:** Emotional Intelligence Scores of Postgraduate Medical Students in Delhi (Overall Scores and Subscales of the Schutte’s Self-Report Emotional Intelligence Test) (n = 200)

	*Overall score*	*Perception of emotions*	*Management of own emotions*	*Management of others’ emotions*	*Utilization of emotions*
Mean Score	124.4	35.9	35.34	29.52	23.55
Standard Deviation	12.81	5.48	4.23	3.64	2.90

The mean EI scores were not found to differ on the basis of gender, marital status, or residential status. There was no significant difference observed between the students by their specialty or year of study. Emotional intelligence scores decreased with an increase in the number of hours worked by the postgraduate students per week (Table 3). A one-way ANOVA with posthoc tests showed a statistically significant association between emotional intelligence and the total number of hours worked per week (p = 0.013). Study participants working 48 hours per week had higher emotional intelligence than those working more than 96 hours per week (p = 0.009). Those residents who did not have any night duty hours or emergency duty had higher emotional intelligence than those who had night duty hours (p = 0.019) and emergency duty (p = 0.037) (Table 3).

**Table 3:** Association Between Emotional Intelligence score and Work-related Characteristics (n = 200) *Association between two factors tested by t-test **Association between three factors tested by one way analysis of variance (ANOVA) ¶Tukey’s posthoc test for ANOVA shows a significant difference in emotional intelligence between students working < 48 hrs per week and > 96 hrs per week (p = 0.009) EI: emotional intelligence; SD: standard deviation

*Factor*	*n*	*Mean EI Score +/- SD*	*p-value*
*Year of Study*			
First Year	68	122.97 ± 13.24	0.361^**^
Second Year	66	124.05 ± 14.08	
Third Year	66	126.09 ± 10.86	
*Total Hours Worked per Week*			
< 48 hours	32	128.66±10.57	0.013^**¶^
48 - 96 hours	145	124.34 ± 13.07	
> 96 hours	23	118.43 ± 12.02	
*Night Duty*			
No	25	129.9 ± 10.85	0.019^*^
Yes	175	123.6 ± 12.9	
*Emergency Duty*			
No	24	129.5 ± 10.6	0.037^*^
Yes	176	123.7 ± 12.9	

## Discussion

The perception, management, and utilization of emotions are essential for the successful career of a doctor. An emotionally intelligent doctor may be able to better perceive the needs of their patients and, in turn, provide better care, leading to improved patient satisfaction [4]. In addition, healthcare, as a whole, is provided by a number of functionaries with the doctor comprising a small part of the team. In the Indian scenario, the burden of leadership and management of the healthcare team falls on the doctor. Emotionally intelligent doctors will be able to better head and manage the healthcare team, finally leading to the provision of better healthcare to the patient [2-3].

Our study measured the emotional intelligence of postgraduate medical students from two government medical colleges in Delhi, using the Schutte’s Emotional Intelligence Scale. The scores are observed to be almost the same as the corresponding scores of university students in India, as measured by the same scale [25].

As the students become older, they become more emotionally intelligent (r = 0.187, p = 0.008). This is in line with the findings of McKinley [2], Weng, et al. [22], Faye, et al. [26], and Zeidner, et al. [27]. Life experiences of a person increase with their age, and as they mature, they become more sensitive to their own feelings and to those of others.

Emotional intelligence is an essential part of the repertoire of doctors, where sensitivity and tact are needed in dealing with patients in their everyday practice. Research has been done to find out if emotional intelligence varies among the different medical and surgical specialties [10, 18, 28]. Our study also supports these findings – there is no significant difference in emotional intelligence between postgraduate students of different specialties. It can be said that specialty does not play a role in the development of EI.

Emotional intelligence has been found to change with the year of study of the resident [11, 29-30]. Our study demonstrates an increasing trend in emotional intelligence scores over time, between postgraduate students of first (122.97 ± 13.24), second (124.05 ± 14.08), and final years (126.09 ± 10.86) (Table 3).

The workload of doctors also affects their emotional intelligence scores. The emotional intelligence of the postgraduate residents was found to decline with the increase in the total number of hours worked per week (p = 0.013). This difference was found to be statistically significant between the residents who worked about 48 hours per week and those who worked more than 96 hours per week (p = 0.009) (Table 3). This finding compared with that of Faye, et al. [26], who reported that residents who worked more than 50 hours per week were less emotionally intelligent.

Emotional intelligence was seen to be lower in those students who had worked night duty hours (p = 0.019) (Table 3). The increase in the total workload of the participants may possibly leave less time for them to spend on recreational activities, relaxation, and sleep. This may ultimately result in the individual being unable to effectively manage their emotions [26].

Emergency duty hours represent a charged, high-tension working environment, where every decision made by both the doctors as well as the healthcare team has life-saving implications. Our study demonstrates a statistically significant difference (p = 0.037) in emotional intelligence between those postgraduate students who had emergency duty (123.7) and those who did not (129.5) (Table 3). Stress, in a moderate amount, will lead to improved performance and better quality of work. However, there seems to be a certain threshold beyond which an increase in stress will cause a drop in performance and ultimately have a negative effect on the individual.

People who have experienced either physical or emotional trauma would have more experience in dealing with negative emotions and managing them. This would thereby reflect in their total emotional intelligence. Findings have been reported by Faye, et al. [26], which state that residents who had some problem at home had a higher self-awareness and sensitivity to suffering. This would eventually affect their bedside manner and patient handling skills. Our study assessed for a history of significant physical and emotional trauma over the past one year. We observed no significant differences between residents who had experienced trauma as compared to those who had not.

Our study observed that the emotional intelligence of postgraduate students who reported a significant source of stress in their life (122.2 ± 12.9) was lower than those residents who did not report such a source of stress in their life (124.8 ± 12.8), but this difference was not significant (Table 4). Emotionally intelligent residents may also be able to better manage the stress in their life. In contrast to residents with low emotional intelligence, emotionally intelligent residents may not perceive similar sources of stress to be significantly severe (i.e., family, work, studies, personal, etc.).

**Table 4:** Association Between Emotional Intelligence and Personal Factors (n = 200) EI: emotional intelligence: SD: standard deviation

*Factor*		*N*	*Mean EI Score +/- SD*	*p-value*
*Traumatic Event*				
Death of a family member	No	174	123.9 ± 13.11	0.186
	Yes	26	127.5 ± 10.23	
Involved in an accident	No	185	124.30 ± 12.72	0.824
	Yes	15	125.0 ± 14.27	
Relationship break-up	No	188	123.9 ± 12.72	0.112
	Yes	12	130.6 ± 13.04	
Other significant trauma	No	195	124.1 ± 12.59	0.065
	Yes	5	134.8 ± 18.17	
*Self-reported History of Psychiatric illness*				
No		196	124.2 ± 12.87	0.261
Yes		4	131.5 ± 6.60	
*Self-reported History of Significant Stress*				
No		165	124.8 ± 12.78	0.274
Yes		35	122.2 ± 12.91	

### Limitations

Being a cross-sectional study, causal associations could not be established between factors and EI. Also, changes in emotional intelligence with time in an individual subject could not be measured. Association with other factors, such as substance abuse, was not measured. This study did not focus on the qualitative aspects of emotional intelligence. Our study focused only on postgraduate students who had an active involvement in patient care. In the future, participants may be selected from non-clinical and paraclinical specialties, and a comparison between these groups might be done.

## Conclusions

The emotional intelligence of postgraduate medical students in Delhi has been measured and has been found to increase with age and experience. It was also observed that emotional intelligence was negatively affected by an increase in the total workload of the resident doctors, having night duties, and having emergency duties. However, it was not affected by factors like gender, religion, place of residence, choice of specialty, year of study, or past history of trauma.
